# USP7 inhibits FOXO1 activity in chronic lymphocytic leukaemia cells enhancing cell survival

**DOI:** 10.1002/1878-0261.70350

**Published:** 2026-10-21

**Authors:** Hassan N.B. Almuhanna, Jodie Hay, Michael W. Moles, Lucy Clarke, Jennifer Cassels, Alison Sefcick, Alison M. McCaig, Heather G. Jørgensen, Alison M. Michie

**Affiliations:** ^1^ School of Cancer Sciences, College of Medicine, Veterinary and Life Sciences University of Glasgow UK; ^2^ NHS Greater Glasgow & Clyde Royal Alexandra Hospital Paisley UK; ^3^ NHS Greater Glasgow & Clyde Queen Elizabeth University Hospital UK

**Keywords:** B‐cell receptor, chronic lymphocytic leukaemia, deubiquitinases, FOXO1, USP7

## Abstract

Signals emanating from the B cell receptor (BCR) are vital for driving cell survival and proliferation in chronic lymphocytic leukaemia (CLL). BCR‐mediated signals in CLL cells promote nuclear exclusion and inactivation of the transcription factor forkhead box protein O1 (FOXO1) and transient upregulation of FOXO1 expression. To investigate the mechanisms that regulate FOXO1 inactivation, we focused on the role of deubiquitinase (DUB) family proteins. Stratifying expression of individual DUB proteins, we established that ubiquitin‐specific protease 7 (USP7), USP8, USP9x, USP10 and USP14 are significantly upregulated compared to healthy B cells. USP7 interacts with FOXO1 in primary CLL cells, suggesting that modulation of USP7 activity may impact FOXO1 function. Using selective USP7 inhibitors, P5091 and HBX19818, or USP7 knockdown, combined with ibrutinib, we demonstrated enhanced FOXO1 nuclear localisation over ibrutinib alone and increased FOXO1 activity, leading to modulation of selected FOXO target genes. This resulted in reduced CLL cell viability and enhanced cell cycle arrest in MEC1 cells, largely reversed by FOXO1 knockdown. These findings suggest that dual targeting of BCR signalling and USP7 activity can harness FOXO1‐mediated anti‐tumour activity and may represent a novel therapeutic approach for CLL treatment.

## Graphical abstract

CLL cells rely on BCR‐mediated signalling for survival and proliferation in part through suppression of FOXO1. FOXO1 activity is regulated by a plethora of post‐translational modifications including phosphorylation and ubiquitination. We demonstrated that dual inhibition of BCR signalling and the deubiquitinase USP7 activates FOXO1, modulating its target genes to promote cell cycle arrest and apoptosis in CLL cells. Created in BioRender. Elmarghany, A. (2026) https://BioRender.com/e6o02lw.

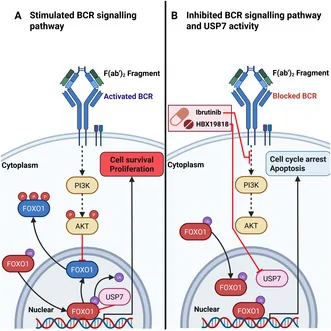

**Abbreviations**
BCR: B‐cell receptor
BTK: Bruton's tyrosine kinase
CLL: chronic lymphocytic leukaemia
DUB: deubiquitinase
FOXO1: forkhead box protein O1
IBR: ibrutinib
KD: knockdown
MNCs: mononuclear cells
mTOR: mammalian target of rapamycin
NDC: no drug control
PB: peripheral blood
PI3K: phosphatidylinositol 3‐kinase
PTEN: phosphatase and tensin
UPS: ubiquitin‐proteasome system
USP: ubiquitin‐specific protease
7AAD: 7‐amino‐actinomycin D

## 1 Introduction

CLL is a malignant disorder characterised by monoclonal expansion and progressive infiltration of CD19^+^CD5^+^IgM^lo^IgD^lo^ B cells in the peripheral blood (PB), bone marrow (BM) and secondary lymphoid organs [1]. The clinical progression of CLL is highly heterogenous: progressive disease requires therapy shortly after diagnosis, while indolent disease may never require treatment [1, 2, 3, 4]. B‐cell receptor (BCR) mediated signals promote CLL cell survival and proliferation, and its importance in CLL pathogenesis is evident through its structure being a key prognostic factor of disease, and the effectiveness of BCR‐targeted therapies [4, 5]. BTK‐ and PI3K‐targeted inhibitors have significantly improved overall survival of CLL patients, thus transforming the clinical management of patients [6, 7]. However, a significant number of patients acquire resistance to current therapies or discontinue treatment due to toxicities, suggesting the need for additional therapies.

FOXO transcription factors belong to the forkhead box (FOX) family, a large group of proteins that share a conserved winged‐helix DNA binding domain. The FOXO family consists of four members, FOXO1, FOXO3a, FOXO4 and FOXO6, which are negatively regulated by the PI3K/AKT pathway, enabling the promotion of survival and proliferation of malignant cells [8, 9]. Constitutive activation of the PI3K/AKT pathway leads to persistent FOXO1 phosphorylation (at T24, S256, S319), driving its cytoplasmic retention and degradation [9]. FOXOs are additionally regulated by acetylation, methylation and ubiquitination [9, 10]. Ubiquitination is a reversible event targeting proteins and, in the case of FOXO proteins, can result in proteasomal degradation, subcellular localisation or activation, identifying a link between the ubiquitin‐proteasome system (UPS) and FOXO function [11, 12, 13]. For example, degradation of FOXO1 by ubiquitination was dependent on PI3K/AKT‐mediated phosphorylation in HepG2 cells [14]. Protein ubiquitination is reversed by deubiquitinase (DUB) enzymes, cleaving the C terminus isopeptide bond of ubiquitin: there are approximately 100 DUB family members encoded by the human genome [15].

BCR‐mediated activation of the PI3K/AKT/mTORC2 axis leads to the phosphorylation of FOXO1 and its association with 14‐3‐3 protein [16, 17, 18, 19]. This association results in the cytoplasmic sequestration and subsequent proteasomal degradation, potentially mediated by the E3 ligase SKP2 through K48‐linked polyubiquitination [20, 21]. In CLL cells, DUB enzymes, particularly USP7, play a crucial role in oncogenesis through the inactivation of PTEN, the negative regulator of PI3K/AKT signalling [22]. Studies have shown that USP7 interacts with FOXO1, FOXO3a and FOXO4 [21, 22, 23, 24]. Hall *et al*. demonstrated that mono‐ubiquitination of FOXO1 promoted its transcriptional activity, while the removal of this monoubiquitin by USP7 reduced FOXO1 occupancy on the promoter sites of its target genes [24]. Despite the accumulated knowledge about FOXO1 regulation by phosphorylation, the role of DUB proteins in regulating FOXO1 expression in CLL and disease pathogenesis has not been explored.

## 2 Materials and methods

### 2.1 Primary cells and cell lines

PB samples were collected from patients with a confirmed diagnosis of CLL, and the experiments were undertaken with the understanding and written informed consent of each subject. The study cohort comprised treatment‐naïve CLL patients and those who had previously received therapy, provided that at least three months had passed since their last treatment. The study methodologies conformed to the standards set by the Declaration of Helsinki and were approved by the West of Scotland Research Ethics Service, NHS Greater Glasgow and Clyde (UK) with all work being carried out in accordance with the approved guidelines (REC Ref: 20/WS/0066). Samples were collected within haematology clinics at NHS‐GG&C and cryopreserved for study use between from October 2010–December 2024. Linked clinical data of prognostic markers of the CLL cohorts were recorded (Table S1). CLL cell purity was ≥ 95% in all cases, determined by flow cytometry. Buffy coat samples were obtained from the Scottish National Blood Transfusion Service (UK; Ref 17 ~ 10). To enrich for healthy B lymphocytes, we used either positive selection‐ human CD20 MicroBeads (Miltenyi Biotec Ltd, Bisley, UK) for gene expression comparison, or negative selection—human Pan B Cell Isolation (Miltenyi Biotec Ltd) for protein expression comparison [16]. Primary B and CLL cells were cultured at 1 × 10^6^·mL^−1^ in RPMI‐1640 containing 10% foetal bovine serum (FBS), 50 U·mL^−1^ penicillin, 50 mg·mL^−1^ streptomycin and 2 mm L‐glutamine (CLL medium; Invitrogen Ltd., Paisley, UK). The MEC1 CLL cell line (provided by Prof. Joseph Slupsky, University of Liverpool; RRID:CVCL_1870) was cultured in DMEM containing 10% FBS, 50 U·mL^−1^ penicillin, 50 mg·mL^−1^ streptomycin and 2 mm L‐glutamine (MEC1 medium). Cells were cultured at 37 °C in a humidified incubator with 5% (v/v) CO_2_. Cell lines were authenticated based on functional behaviour, morphology and/or phenotype, were tested for mycoplasma contamination every 6–8 months, and were mycoplasma‐free for all experiments.

### 2.2 Cell stimulation and drug treatments

Inhibitors—Ibrutinib (IBR; BTK inhibitor), selective USP7 inhibitors (P5091, HBX19818) and pan DUB inhibitor (PR‐619) were dissolved in DMSO (Stratech Scientific Ltd, Ely, UK). The inhibitors were freshly prepared and diluted in complete RPMI medium (Table S2). DMSO was used as a vehicle control or no drug control (NDC). Soluble αIgM F(ab')_2_ fragments (Stratech Scientific Ltd) were used to crosslink the BCR (BCR‐crosslinking; 10 μg·mL^−1^; 0.5–48 h), incubated at 37 °C. Primary CLL cells (0.7–1 × 10^7^/condition) were pre‐treated with indicated inhibitors or DMSO/vehicle control for 30 min prior to BCR stimulation or left unstimulated (US/control).

### 2.3 Co‐immunoprecipitation (Co‐IP)

The immunoprecipitation (IP) Pierce Crosslink IP kit was used according to the manufacturer's instructions (Thermo Fisher Scientific). In brief, primary CLL cells (10–15 × 10^6^/condition) or MEC1 cells (2–4 × 10^6^/condition) were incubated in ice‐cold IP lysis buffer (0.025 M Tris, 0.15 M NaCl, 0.001 M EDTA, 1% NP‐40, 5% glycerol; pH 7.4 containing protease and phosphatase inhibitors; 10 : 1 volume/weight of cell pellet) for 10 min on ice, generating the whole cell lysate (WCL). Lysates were pre‐cleared with 80 μL agarose resin slurry. The concentration of antibodies (FOXO1, USP7, IgG; Table S3) was optimised to 2 μg for a total protein (500 μg/condition). The antibodies were crosslinked with disuccinimidyl suberate (DSS) to protein A/G plus agarose (ThermoFisher Scientific). Elution buffer (50 mL, pH 2.8, containing primary amine) was used to elute co‐IP protein. The eluted proteins were neutralised with 1 M Tris (pH = 9.5) and prepared for SDS/PAGE. An aliquot of the WCL served as a control.

### 2.4 Lentiviral knockdown

USP7 or FOXO1 shRNA‐mediated knockdown (KD) was performed via lentiviral delivery plasmid using the pLKO.1‐puro and TRC2‐pLKO‐puro constructs (Sigma‐Aldrich; Table S4). Lentiviral particles were generated by transfecting HEK293T cells with VSV‐G (envelope vector), HIV‐1 (packaging vector) and USP7 or FOXO1 shRNA or scrambled (SCR) vector control, using the established calcium phosphate method. Viral particles were cultured with freshly passaged 1 × 10^6^ MEC1 cells in complete DMEM media with 10 μg·mL^−1^ polybrene (Sigma‐Aldrich). After transduction, the cells were resuspended in complete DMEM media with 2 μg·mL^−1^ puromycin to enable puromycin selection, then 1 μg·mL^−1^ puromycin to maintain KD selectivity. KD efficiency was tested using qPCR.

### 2.5 Western blotting

Protein lysates were prepared in 30–50 μL lysis buffer (20 mm Tris pH 7.4, 2 mm EDTA, 1% Triton, 1 mm DTT) containing protease inhibitor cocktail and phosphatase inhibitor cocktail (Roche) and incubated on ice for 30 min. The concentration of protein lysates was determined using bicinchoninic acid assay (BCA) protein assay kit (Pierce, ThermoFisher Scientific), following the manufacturer instructions. When phospho‐ and total proteins were analysed together, mirror blots of the same samples were run at the same time (e.g. Fig. S1A). Protein electrophoresis was performed using NuPAGE® Novex® pre‐cast 4–12% Bis‐Tris gels with Invitrogen Xcell SureLockTM Mini cell system and all the buffers used were purchased from Invitrogen. PVDF membranes were blocked with 5% non‐fat milk in Tris‐buffered saline with 0.1% Tween® 20 detergent (TBST) (10 mm Tris/HCl, 50 mm NaCl, 0.1% Tween 20 (pH7.4)). List of antibodies are listed in Table S3. Immunodetection was carried out using the Immobilon® Forte Western HRP Substrate (Sigma) by Li‐Cor Odyssey® and were further analysed in image studio™ lite v5.2 software with densitometry carried out following the manufacturer instructions.

### 2.6 Flow cytometry

For apoptosis analysis, 5–10 × 10^5^ treated cells were washed and pelleted in HBSS (ThermoFisher Scientific) at RT. Cells were incubated in 100 μL HBSS containing 2.5 μL Annexin V and 2.5 μL 7AAD (BD Biosciences) for 15 min at RT in the dark and then quenched with 200 μL HBSS before analysis. For cell proliferation, cells were stained with CellTrace™ Violet stock solution (Invitrogen, Paisley, UK) as per the manufacturer's protocol. MEC1 cells were passaged and then seeded into appropriate plate at (1 × 10^6^/condition). For cell cycle analysis, 1 × 10^6^ treated MEC1 cells were fixed and permeabilized by adding 1 mL cold 80% ethanol dropwise to the pellet. After washing the samples, 400 μL propidium iodide (PI)/RNAse Staining Buffer (BD Biosciences) was added and the cells were incubated at RT in the dark for 15 min before analysis. All data were acquired on a FACSCantoII with BD facsdiva software and analysed using flowjo software (Tree Star Inc., OR).

### 2.7 Cellular fractionation

The nuclear and cytoplasmic protein were generated by using Nuclear Extraction Kit (Active Motif), with 7–10 × 10^6^/condition for primary CLL cells or 1–2 × 10^6^/condition for MEC1 cells [25]. An aliquot of WCL was used as a control. The cells were mildly lysed with 50 μL 1× hypotonic buffer for 15 min on ice, followed by the addition of detergent at a ratio of 1 : 20 of hypotonic buffer and vortexed for 10 s to generate the cytoplasmic fraction. The nuclear fraction was then generated by adding 50 μL complete lysis buffer and incubating for 30 min on ice. Protein lysates were denatured and immunoblotted as described.

### 2.8 FOXO1 activity

FOXO1 transcription factor activity was quantified using a DNA‐binding ELISA 96 plate TransAM® FKHR (FOXO1) Transcription Factor ELISA Kit (Active Motif), using the nuclear fraction of cells [16]. The FOXO1 activity kit was followed according to the manual provided by the manufacturer. The protein content of the nuclear fraction was quantified, and an equal concentration (10–15 μg) was used for all samples. The absorbance was read at 450 nm using the SpectraMax M5 microplate reader (Molecular Devices).

### 2.9 qRT‐PCR

RNA was extracted using the RNA‐Easy mini kit following the manufacturer's instructions (QIAGEN). cDNA synthesis was prepared with the SuperScriptTM III Reverse Transcriptase kit (ThermoFisher Scientific) using 500 ng/condition of RNA. qPCR was carried out using a TaqMan® 2× Universal PCR Master Mix (ThermoFisher Scientific), on a MicroAmpTM Optical 384 reaction plate in triplicate, and run on the 7900HT Fast Real‐Time PCR system (Applied Biosystems, Warrington, UK), programmed to complete 40 cycles. Each cycle consisted of 50 °C for 2 min, 95 °C for 10 min, 95 °C for 15 s and 60 °C for 1 min. The results were analysed using sequence detection systems software v 2.3 (Applied Biosystems). The cycle threshold (Ct) was calculated for reference gene and target gene. The results were expressed as ∆∆Ct. The primers used are listed in Table S5.

### 2.10 Statistics

Data were analysed using graphpad prism software v9.4.1 (GraphPad Software Inc., San Diego, CA). *P* values were determined by two‐tailed Students' paired or unpaired *t*‐test or mixed model ANOVA on a minimum of at least 3 biological replicates as indicated in the figure legends. Normality test (Shapiro Wilk test) was applied due to *t*‐test and ANOVA test assumption of sample normality. Mean *±* standard error of mean (SEM) is shown.

*P ≤ 0.05; **P ≤ 0.01; ***P ≤ 0.001; ****P ≤ 0.0001.

## 3 Results

### 3.1 FOXO1 expression is transiently upregulated in a BCR‐dependent manner

Analysis of FOXO1 phosphorylation (p‐FOXO1^T24^) and expression in PB‐derived CLL samples revealed that basal p‐FOXO1^T24^ was significantly downregulated and FOXO1 protein level was significantly upregulated in CLL cells, compared to healthy B cells (Fig. 1A,B; Fig. S1A,B). Stratification of p‐FOXO1^T24^ based on cytogenetic abnormalities revealed a significant upregulation of p‐FOXO1^T24^ in CLL cells with poor prognostic characteristics, while FOXO1 expression showed no significant difference (Fig. S1C). In agreement with our previous findings, we show that FOXO1 is upregulated in CLL cells [16], and is more inactive in poor prognostic patients.

**Fig. 1:**
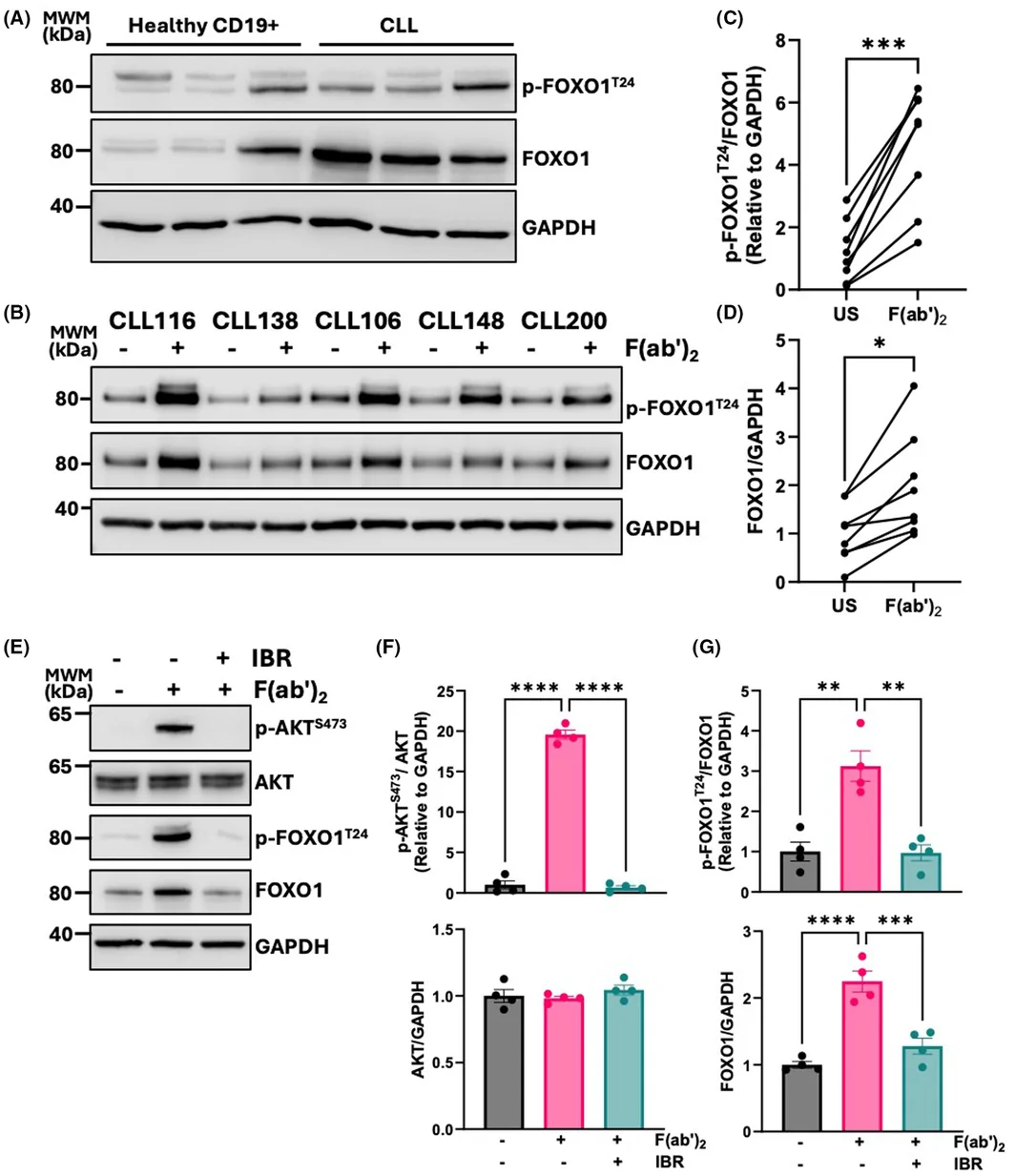
FOXO1 expression is increased rapidly in primary CLL cells short term in a BCR‐mediated manner. (A) Representative western blot to assess FOXO1 expression/activation in *ex vivo* PB‐derived CLL cells (*n* = 19) compared with healthy CD19^+^ cells from healthy donors (*n* = 6). The blot was probed for p‐FOXO1^T24^, FOXO1, and GAPDH (loading control). Densitometry is shown in Fig. S1; (B). Representative western blot of primary CLL samples as indicated, BCR‐crosslinking with F(ab')_2_ fragments (10 ng·mL^−1^) for 30 min (+) or left unstimulated (US; −). The blot was probed for p‐FOXO1^T24^, FOXO1, and GAPDH (loading control). Levels of p‐FOXO1^T24^/FOXO1 (C) and FOXO1 (D) relative to GAPDH in US and BCR‐crosslinked cells (*n* = 8) are shown. (E) Representative western blot of BCR‐crosslinked primary CLL sample (F(ab')_2_ fragments–10 ng·mL^−1^) for 30 min (+) alone or in the presence of ibrutinib (IBR; 1 μm) or left US (−). The blot was probed for p‐AKT^S473^, AKT, p‐FOXO1^T24^, FOXO1, and GAPDH (loading control). Levels of p‐AKT^S473^/AKT and AKT (F) and p‐FOXO1^T24^/FOXO1 and FOXO1 (G) are shown relative to GAPDH in US and BCR‐crosslinked cells (*n* = 4) are shown. Each dot represents an individual CLL sample. Data expressed as the mean ± SEM. Statistics calculated by paired *t*‐test; *P < 0.05, **P < 0.01, ***P < 0.001, ****P < 0.0001.

BCR crosslinking of CLL cells resulted in a significant upregulation of p‐FOXO1^T24^ as expected, together with an elevation in FOXO1 expression within 0.5 h in all CLL patient cells assessed (Fig. 1B–D). Analysis of the kinetics over 24 h showed that p‐AKT^S473^ was maximally upregulated at 0.5 h before reducing and reaching basal levels at 4 h (Fig. S2A,B). The p‐FOXO1^T24^ levels reached maximal levels between 0.5–1 h, reducing to basal levels by 4 h (Fig. S2A,C). FOXO1 levels were significantly upregulated at 0.5–1 h in BCR‐crosslinked CLL cells compared to unstimulated cells, before reducing to basal level beyond 2 h (Fig. S2A,C). AKT expression did not change with BCR stimulation. *FOXO1* levels remained unaltered across the time‐course of BCR‐crosslinking (Fig. S2D), suggesting the upregulation of FOXO1 is post‐translational.

The BCR‐mediated elevation in FOXO1 expression was reduced to basal levels, together with p‐AKT^S473^ and p‐FOXO1^T24^, when primary cells were treated with ibrutinib (Fig. 1E–G), and no changes in AKT levels occurred upon BCR‐crosslinking or ibrutinib treatment (Fig. 1F). The BCR‐mediated elevation in FOXO1 expression was also reduced in primary cells when mTOR kinase or AKT were targeted with AZD8055 or AZD5383 respectively (Fig. S3A,B). These data were supported by data generated in MEC1 cells, a CLL cell line that expresses an unmutated *IGHV*, resulting in constitutive BCR signalling and high basal phosphorylation of AKT/FOXO1 [26]. Ibrutinib treatment resulted in a significant and sustained downregulation of p‐AKT^S473^ and p‐FOXO1^T24^ at 1 and 24 h compared to untreated MEC1 cells (Fig. S4A–C). FOXO1 levels were significantly downregulated at 1 h of treatment with ibrutinib compared to untreated MEC1 cells, similar to that observed in primary CLL cells (Fig. S4C). However, by 24 h, FOXO1 expression returned to MEC1 baseline levels. AKT protein levels were unaffected by ibrutinib treatment at both timepoints (Fig. S4B). These data demonstrate the utility of MEC1 cells for investigating the mechanism relating to the short‐term BCR‐mediated FOXO1 upregulation in CLL cells.

### 3.2 Expression of selective DUB family members are modulated in primary CLL cells

Given the rapid upregulation of FOXO1 expression downstream of BCR crosslinking, and previous reports of USP7 regulating FOXO family members [21, 22], we examined DUB family members' expression and regulation of FOXO1. The STRING database indicated that FOXO1 expression is associated with USP7, which in turn links by co‐expression with USP8, USP9x, USP10, USP14 and USP28, all of which have been connected to haematological malignancies [27, 28, 29, 30] (Fig. 2A). Assessment of these DUB proteins in CLL cells showed that USP7 was significantly upregulated in CLL cells compared with healthy controls, independent of Binet stage (Fig. 2B). Similarly, significant upregulation of USP8, USP9x, USP10 and USP14 expression was observed in CLL cells, with a significant increase in USP9x observed in Binet stage B CLL patients (Fig. S5A–F). USP28 was only slightly elevated in CLL patient cells compared to healthy controls (Fig. S5G).

**Fig. 2:**
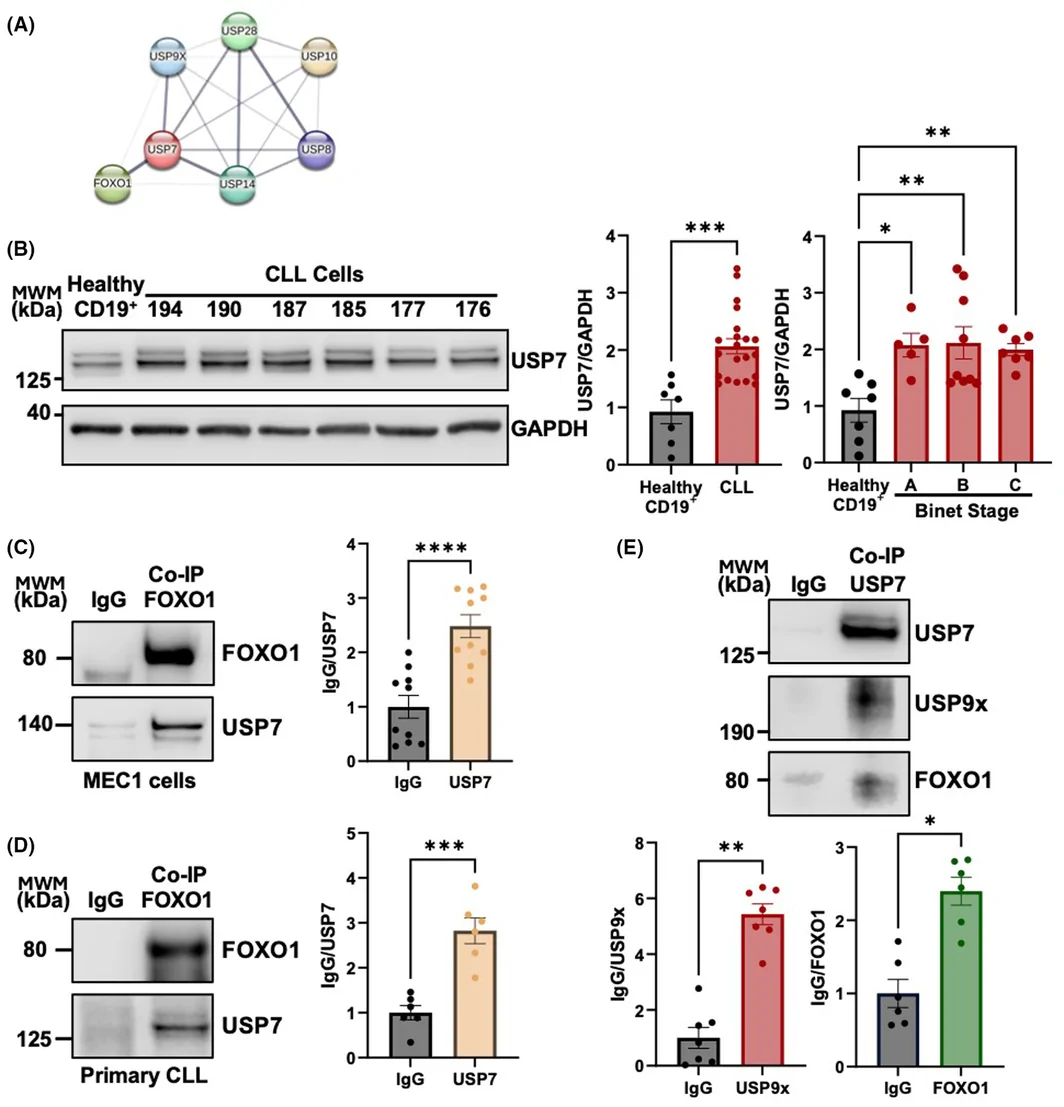
USP7 associates with FOXO1 in primary CLL cells. (A) Protein–protein network interaction map was generated and downloaded from STRING database website (2021). (B) Representative western blot to assess protein expression of *ex vivo* PB‐derived CLL cells compared with healthy CD19^+^ cells from healthy donors, probed for USP7 and GAPDH (loading control; same blot as shown in Fig. S1A). Lower left: Relative protein expression of USP7 relative to GAPDH, between B cells from healthy donors (Healthy CD19^+^; *n* = 7) and CLL patient samples (CLL; *n* = 21). Lower right: Relative protein expression of USP7 relative to GAPDH between primary CLL patient samples is shown, comparing healthy CD19^+^ with CLL samples by Binet stage (*n* ≥ 5); (C) Representative western blot of FOXO1 co‐immunoprecipitation (co‐IP) in MEC1 cells (or IgG control) subsequently probed for FOXO1 and USP7. The relative levels of proteins pulled down in FOXO1 co‐IP compared to IgG control for USP7 (*n* = 10); (D) Representative western blot of FOXO1 IP in primary CLL cells (CLL204; or IgG control) subsequently probed for FOXO1 and USP7. The relative levels of proteins pulled down in FOXO1 co‐IP compared to IgG control for USP7 (*n* = 6); (E) Representative western blot of USP7 immunoprecipitation (or IgG control) in MEC1 cells probed for USP7, USP9x and FOXO1. The relative levels of proteins co‐immunoprecipitated with USP7 compared to IgG control are shown for USP9x (left; *n* = 7) and FOXO1 (right; *n* = 6). The mean expression levels of proteins in IgG control were used to calculate the relative protein expression levels. Each dot represents an individual CLL sample. Data expressed as the mean ± SEM. Statistics calculated by paired (B) or unpaired (C–E) *t*‐test; *P < 0.05, **P < 0.01, ***P < 0.001, ****P < 0.0001.

Performing co‐IPs, we established that FOXO1 interacts with USP7 compared to IgG controls in MEC1 and primary CLL cells (Fig. 2C,D). However, FOXO1 did not co‐IP with other DUB family members analysed: USP8, USP9x, USP10 and USP14 (data not shown). The reciprocal interaction revealed a significant interaction between USP7 and both USP9x and FOXO1 compared to IgG controls (Fig. 2E), while USP8 and USP14 were not detected with USP7 IP in MEC1 cells (data not shown). These studies demonstrate a FOXO1‐USP7 interaction in CLL cells.

### 3.3 USP7 KD enhances FOXO1 nuclear translocation and activity in MEC1 cells

To assess the role of USP7 in regulating FOXO1 activity, MEC1 cells were transduced with shRNA lentiviral vectors targeting *HAUSP/USP7*. USP7 gene and protein expression levels were significantly reduced by construct USP7‐78 (42% reduction in protein expression) compared to SCR control (Fig. 3A,B). Interestingly, USP7 KD significantly upregulated p‐AKT^S473^ and p‐FOXO1^T24^ levels compared to SCR controls, which were inhibited with ibrutinib treatment (Fig. 3C; Fig. S6A,B). While AKT expression was not altered by USP7 KD or ibrutinib treatment, FOXO1 expression was significantly elevated in USP7 KD cells, leading to an enhanced reduction in FOXO1 expression upon ibrutinib treatment in USP7 KD cells compared with the SCR control (Fig. 3C; Fig. S6C,D).

**Fig. 3:**
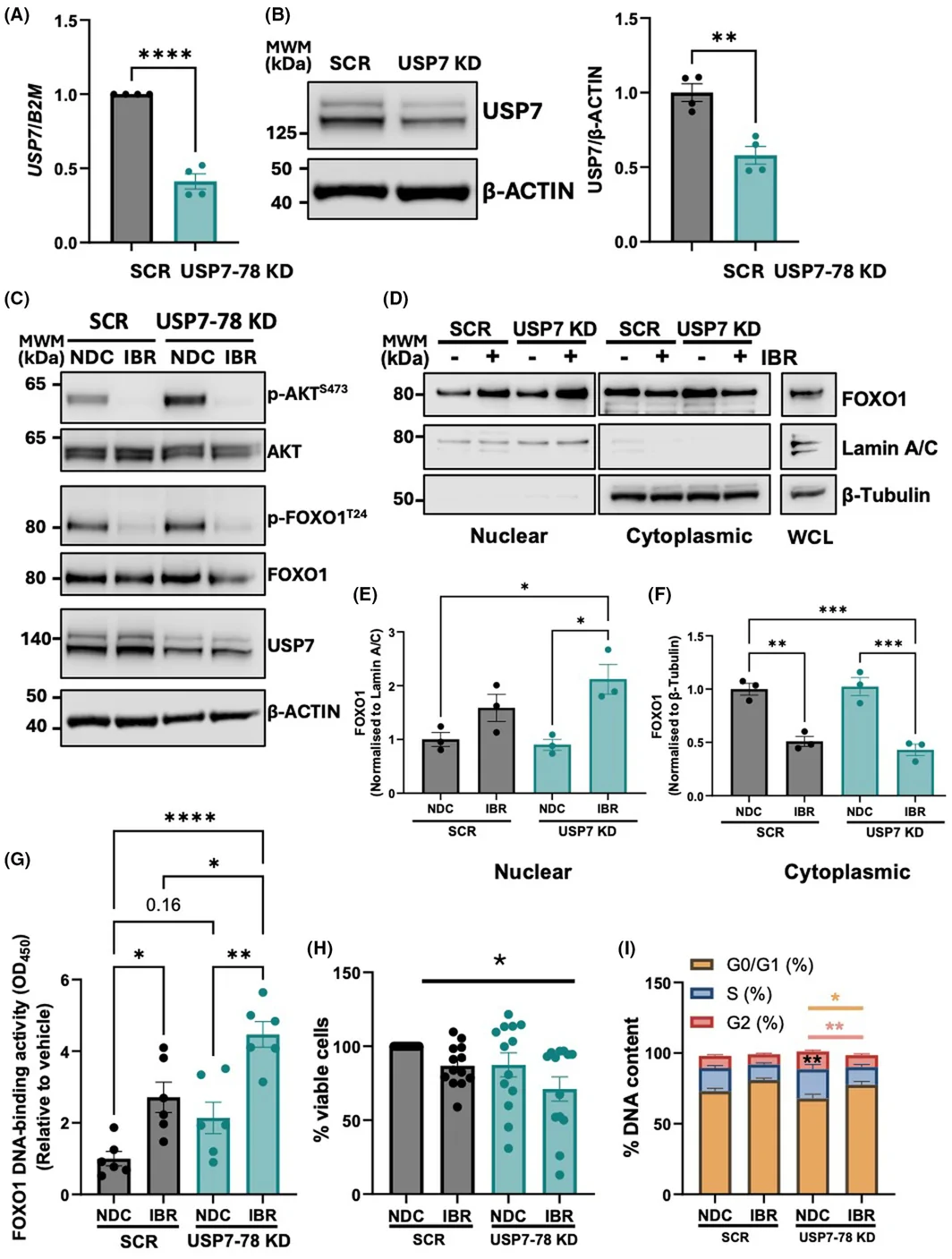
USP7 knockdown leads to an upregulation in FOXO1 activity in MEC1 cells. MEC1 cells were lentivirally‐transduced with USP7‐targeting shRNA knockdown (KD) constructs or scrambled (SCR) control. (A) RT‐qPCR of *USP7* (*n* = 4) relative to *B2M* (reference gene) is shown in USP7‐78 KD MEC1 cells compared to SCR control (*n* = 4); (B) Representative western blot of MEC1 transduced with shRNA‐USP7‐78 and SCR control. The blots were probed with USP7 and β‐ACTIN (loading control). Relative protein levels of USP7 and SCR between USP7 KD and SCR MEC1 cells (*n* = 4); (C) Representative western blot of MEC1 transduced with shRNA USP7‐78 or SCR constructs (*n* = 9). The blots were probed with p‐AKT^S473^, AKT, p‐FOXO1^T24^, FOXO1, USP7, and β‐ACTIN (loading control). Densitometry is shown in Fig. S6; (D) Representative western blot of subcellular fractionation of MEC1 cells transduced with shRNA USP7‐78 KD or scrambled (SCR) control, both treated with ibrutinib (IBR; 1 μm) or left untreated (NDC/DMSO). Following 1 h drug treatment, cells (*n* = 3) were fractionated into cytoplasmic and nuclear fractions or left as whole cell lysates (WCL). The blots were probed for FOXO1, Lamin A/C (nuclear loading control), and β‐Tubulin (cytoplasmic loading control). Protein expression of (E) nuclear FOXO1, and (F) cytoplasmic FOXO1 is shown, relative to appropriate loading controls; (G) Nuclear fractions were generated by subcellular fractionation of USP7‐78 or SCR MEC1 cells treated with IBR (1 μm) for 1 h. The TransAM FOXO1 activity assay was used to determine FOXO1 DNA‐binding activity (*n* = 6); (H) USP7‐78 or SCR MEC1 cells were stained with Annexin V and 7‐AAD to assess cell viability following treatment with IBR (1 μm) for 48 h, or no drug control (NDC/DMSO). Viability is defined as Annexin V negative and 7‐AAD/DAPI negative (*n* = 13). Percentage viable cells for each condition are relative to SCR vehicle; (I) USP7‐78 or SCR MEC1 cells were treated with IBR for 72 h, or NDC/DMSO, then the cell cycle phases were analysed with flow cytometry using PI. After excluding dead cells (by FSC/SSC gating and sub‐G0/G1 exclusion), cell cycle phases were gated. Percentages of each phase G0/G1 (orange), S (blue), and G2/M (red) were plotted for SCR or KD cells in the presence and absence of IBR treatment (*n* = 10 each). Data are the same as shown in Fig. S8. Each dot represents an individual MEC1 biological replicate. Data expressed as the mean ± SEM. Statistics calculated using one‐way ANOVA Tukey test; *P < 0.05, **P < 0.01, ***P < 0.001, ****P < 0.0001.

USP7 KD alone did not affect nuclear or cytoplasmic FOXO1 levels compared to SCR control cells; however, USP7 KD sensitised FOXO1 nuclear localisation to ibrutinib treatment, with USP7 KD resulting in a significant increase in nuclear FOXO1, exceeding the levels observed in ibrutinib‐treated SCR cells. This was mirrored by a decrease in FOXO1 expression in the cytoplasm (Fig. 3D–F). To quantify FOXO1‐DNA binding, equating to FOXO1 activity, we performed a TransAM FOXO1 activity assay. USP7 KD alone in MEC1 cells resulted in a modest increase in FOXO1 transcriptional activity compared to SCR cells (*P* = 0.16), potentially relating to the elevation in FOXO1 expression (Fig. S6D). FOXO1 activity was significantly enhanced with ibrutinib treatment in both the SCR and USP7 KD MEC1 cells. Notably, there was an enhanced increase in FOXO1 activity in USP7 KD cells in the presence of ibrutinib compared with SCR cells, indicating that USP7 diminishes ibrutinib‐mediated FOXO1 activation (Fig. 3G). The elevation in FOXO1 activity resulted in increased transcription of FOXO1 target genes with a significant elevation in *FOXO4, CDKN1A, BCL2L11* and *IGFR1* expression in ibrutinib‐treated USP7 KD MEC1 cells (Fig. S7A–H). Assessing apoptosis and cell cycle, USP7 KD cells were more sensitive to ibrutinib treatment, significantly reducing cell viability in USP7 KD cells compared to untreated USP7‐KD cells, and significantly increasing cell cycle arrest when treated with ibrutinib. These results suggest that USP7 KD potentiates apoptosis and cell cycle arrest in the presence of ibrutinib (Fig. 3H,I; Fig. S8A–C).

### 3.4 Ibrutinib combined with DUB inhibitors enhances cell cycle arrest and apoptosis in CLL cells

To assess the selectivity of DUB inhibitors towards CLL cells, we treated mononuclear cells (MNCs) from healthy individuals, primary CLL cells and MEC1 with increasing concentrations of PR‐619 (pan‐DUB inhibitor) and two USP7 selective inhibitors (P5091, HBX19818; Figs S9, S10). PR‐619 and P5091 reduced viability in a concentration‐dependent manner, with a lower IC_50_ for CLL cells compared with MNCs (Fig. S9A–G). These data indicated that PR‐619 and P5091 selectively targeted CLL cells for apoptosis. HBX19181 reduced MEC1 cell viability in a concentration‐dependent manner, similar to that of P5091 (Fig. S10A–D). All inhibitors displayed a lower IC_50_ for primary B cells compared to T cells, suggesting a sensitivity of B lineage cells to DUB inhibitors. As expected, PR‐619 treatment of MEC1 cells resulted in a rapid and dose‐dependent elevation in global protein ubiquitination (Fig. S11).

To investigate the combination of ibrutinib with DUB inhibitors in CLL cells, we treated MEC1 cells with PR‐619, P5091 or HBX19818 alone or in combination. Ibrutinib combined with each DUB inhibitor resulted in further reduction in viability, which was significantly reduced compared to ibrutinib alone in each case (Fig. 4A). Similarly, DUB inhibitors significantly reduced MEC1 proliferation, which was further enhanced when combined with ibrutinib, supporting the PI analysis (Fig. 4B,C). P5091 or HBX19818 alone or in combination reduced MEC1 cell cycling, with ibrutinib and HBX19818 significantly modulating the G1/G0 and S phases, while P5091 treatment caused a significant reduction in S phase only (Fig. 4C; Fig. S12A–F). Of note, HBX19818 combined with ibrutinib caused a significant reduction in the viability of primary CLL cells compared to ibrutinib alone, and further reduced viability of CLL cells over HBX19818 treatment alone (Fig. 4D), mirroring the MEC1 cell data.

**Fig. 4:**
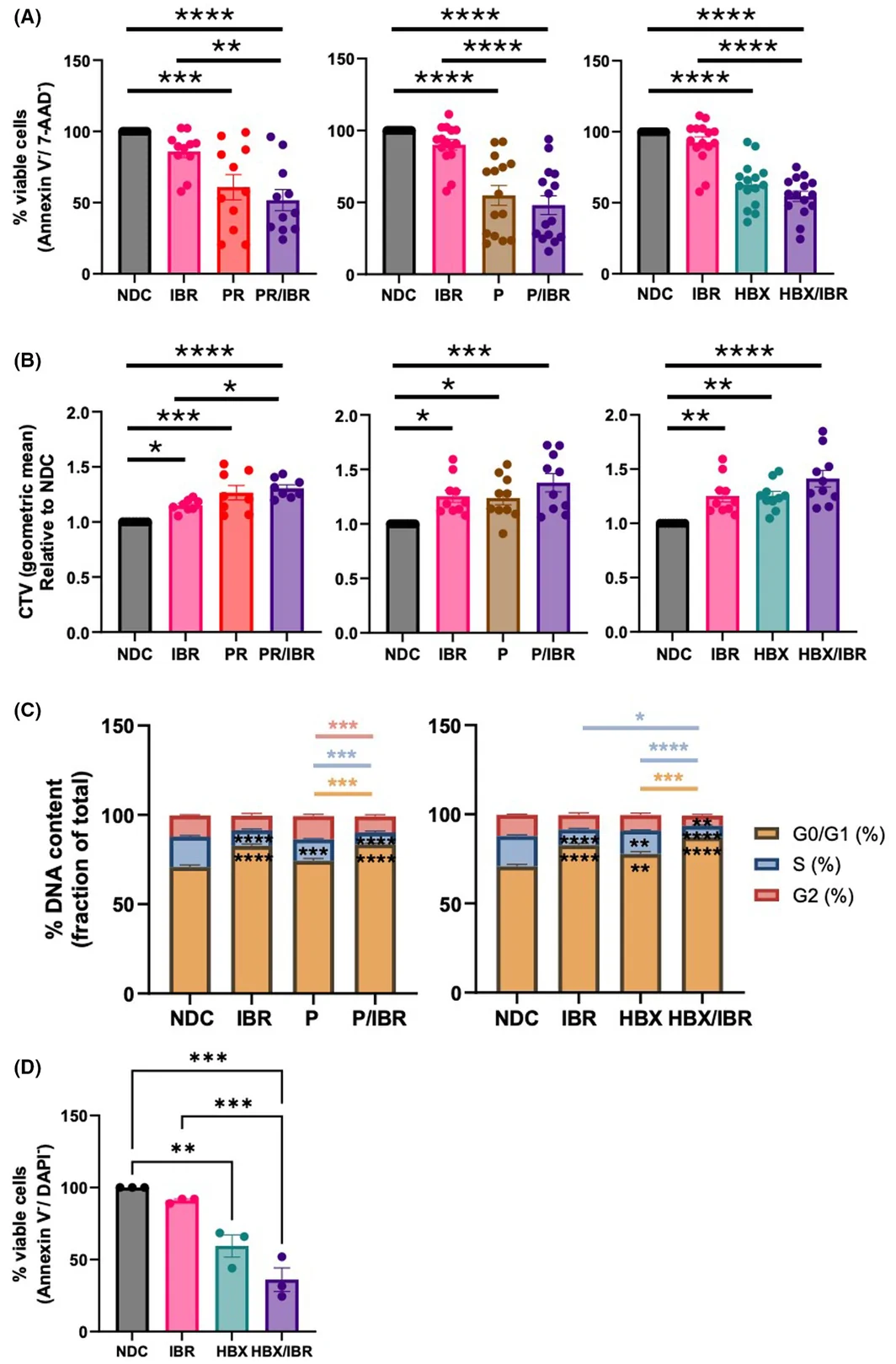
Apoptosis is enhanced when ibrutinib is combined with DUB inhibitors in MEC1 cells. (A) MEC1 cells were treated with IBR (1 μm (*n* = 11 (PR)/15 (P)/15 (HBX)), PR‐619 (PR (*n* = 11); 3 μm; left), and combination (PR/IBR (*n* = 11); left), P5091 (P;10 μm (*n* = 15); middle), and combination (P/IBR (*n* = 15); middle), or HBX19818 (HBX (*n* = 15); 10 μm; right), and combination (HBX/IBR (*n* = 15); right) for 48 h. MEC1 cells were stained with Annexin V and 7‐AAD to assess cell viability following treatment with drugs as indicated for 48 h, or no drug control (NDC/DMSO). Viability is defined as Annexin V^neg^ and 7‐AAD/DAPI^neg^. Percentage viable cells for each condition are relative to NDC; (B) MEC1 cells were stained with CTV to evaluate cell proliferation, following drug treatment as indicated for 48 h, or NDC. PR associated treatments (*n* = 8); P and HBX treatments (*n* = 10). Geometric means for each condition are relative to NDC; (C) MEC1 cells were stained with PI for cell cycle analysis to quantify DNA content following drug treatment as indicated, or NDC for 72 h (*n* = 4). Quantification of DNA content in percentage (%) for G0/G1 (orange), S (blue) and G2/M (red). Separate bar graphs of the individual cell cycle phases are shown in Fig. S12; (D) Primary CLL cells were treated with IBR (1 μm), HBX (8 μm) alone and in combination (HBX/IBR) for 48 h (*n* = 3) and then stained with Annexin V and 7‐AAD to assess cell viability. Each dot represents an individual biological replicate. Data expressed as the mean ± SEM. Statistics calculated using one‐way ANOVA Tukey test, with black asterisks indicating statistical significance compared with NDC in C; *P < 0.05, **P < 0.01, ***P < 0.001, ****P < 0.0001.

### 3.5 DUB inhibitors enhanced ibrutinib‐mediated FOXO1 activation in primary CLL cells

To consolidate our findings that ibrutinib treatment of USP7 KD cells augmented nuclear FOXO1 localisation in MEC1 cells (Fig. 3D–F), we investigated the impact of PR‐619 or HBX19818 treatment on FOXO1 localisation/activation in BCR stimulated primary CLL cells. The p‐AKT^S473^, p‐FOXO1^T24^ levels and FOXO1 expression were not altered with inhibitors in the absence of BCR‐crosslinking (Fig. S13A). Upon BCR crosslinking, PR‐619 or HBX19818 significantly inhibited p‐AKT^S473^ levels, although not to the level induced by ibrutinib. The inhibition was further enhanced in combination, compared to BCR‐crosslinked‐NDC levels. Similar patterns of inhibition were seen with p‐FOXO1^T24^ and FOXO1, with the PR‐619 or HBX19818 treatments alone showing a trend towards significance in reducing FOXO1 expression (Fig. S13B,C). As shown previously, BCR‐crosslinking of primary CLL cells resulted in FOXO1 relocating from the nucleus to the cytoplasm (Fig. 5A,B). Unstimulated CLL cells displayed no significant changes in nuclear FOXO1 levels upon ibrutinib, PR‐619, or HBX19818 treatment compared to untreated controls (Fig. 5B‐left). BCR‐crosslinked CLL cells exhibited a significant downregulation of cytoplasmic FOXO1 with ibrutinib treatment compared to untreated controls. While PR‐619 or HBX19818 alone did not alter cytoplasmic FOXO1 levels, combination with ibrutinib downregulated cytoplasmic FOXO1 levels in BCR‐crosslinked CLL cells, with a reduction in each combination compared with stimulated/untreated cells. The changes of FOXO1 location in the cytoplasm with BCR crosslinking and drug treatments were mirrored by opposing significant changes in nuclear FOXO1 levels (Fig. 5B‐middle & right).

**Fig. 5:**
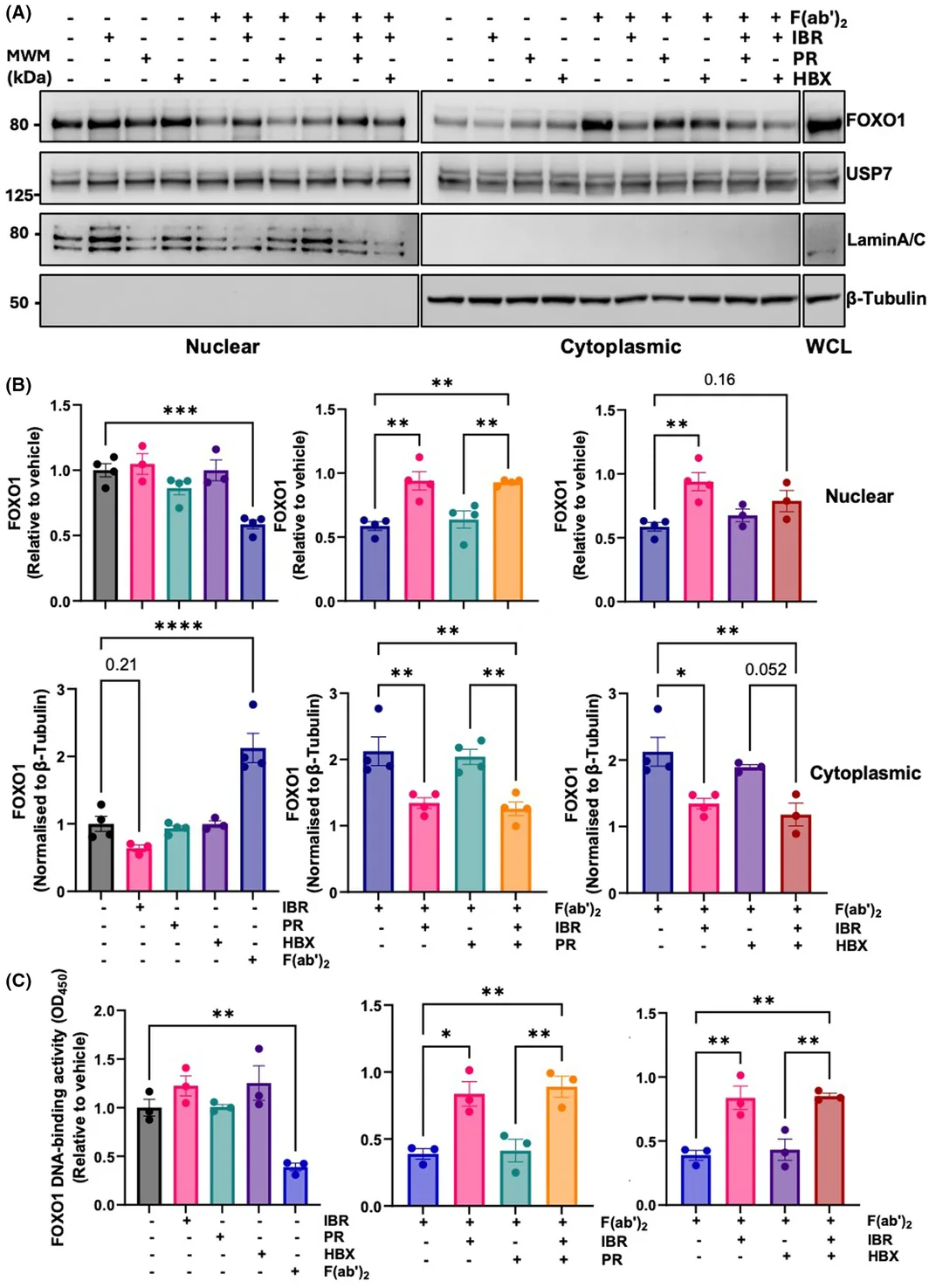
Ibrutinib combined with DUB inhibitors enhanced the effect of ibrutinib in increasing FOXO1 activity in BCR‐stimulated primary CLL cells. Primary CLL cells were BCR crosslinked (10 ng·mL^−1^) for 30 min, following pre‐treatment with ibrutinib (IBR; 1 μm), PR‐619 (PR; 1.5 μm), or HBX19818 (HBX; 8 μm) for 1 h alone or in combination with IBR (1 μm), or left unstimulated. CLL samples (*n* ≥ 3) were then fractionated into cytoplasmic, nuclear, and whole cell lysate (WCL). (A) Representative western blot of subcellular fractionation of primary CLL cells (CLL180) after treatment. The blots were probed for FOXO1, USP7, Lamin A/C (nuclear loading control), and β‐Tubulin (cytoplasmic loading control). (B) Protein expression of (upper) nuclear FOXO1 relative to Lamin A/C or (lower) cytoplasmic FOXO1 relative to β‐Tubulin are shown; Left: FOXO1 levels in unstimulated drug‐treated CLL cells are shown alongside BCR‐crosslinked (F(ab')_2_ stimulated) and NDC/DMSO cells; Middle: FOXO1 levels in F(ab')_2_‐stimulated drug‐treated cells (PR‐619 combinations); Right: FOXO1 levels in F(ab')_2_‐stimulated drug‐treated cells (HBX combinations). (C) FOXO1 activity was analysed from the same CLL cell nuclear fractions as prepared above, using the TransAM FOXO1 activity assay. Left: FOXO1 DNA binding activity in unstimulated drug‐treated CLL cells are shown alongside F(ab')_2_ stimulated and NDC/DMSO cells; Middle: FOXO1 DNA binding activity in F(ab')_2_‐stimulated drug‐treated cells (PR‐619 combinations); Right: FOXO1 DNA binding activity in F(ab')_2_‐stimulated drug‐treated cells (HBX combinations). Each dot represents an individual CLL sample. Data expressed as the mean ± SEM. Statistics calculated using one‐way ANOVA Tukey test, relative to unstimulated NDC; *P < 0.05, **P < 0.01, ***P < 0.001, ****P < 0.0001.

The decrease in FOXO1 DNA‐binding activity seen in BCR‐crosslinked CLL cells, compared to unstimulated controls, was alleviated in the presence of ibrutinib treatment (Fig. 5C). While PR‐619 and HBX19818 treatments alone in stimulated CLL cells did not alter FOXO1 activity, ibrutinib combined with PR‐619 enhanced FOXO1 activity compared to either inhibitor alone (Fig. 5C), suggesting that DUB activity was responsible for reducing FOXO1 activity. To determine whether these changes resulted in modulation of FOXO1 target genes, and focusing on more selective inhibition of USP7, we investigated gene expression changes in HBX19818‐treated CLL cells. Ibrutinib did not alter *FOXO1* expression compared to untreated controls, while HBX19818 alone significantly increased *FOXO1*, which was maintained in combination with ibrutinib in MEC1 cells, but remained unchanged in primary CLL cells (Fig. 6; Fig. S14A). *FOXO4* expression increased in MEC1 cells with HBX19818‐containing treatments, similar to that noted in USP7 KD MEC1 cells (Figs S7C, S14B).

**Fig. 6:**
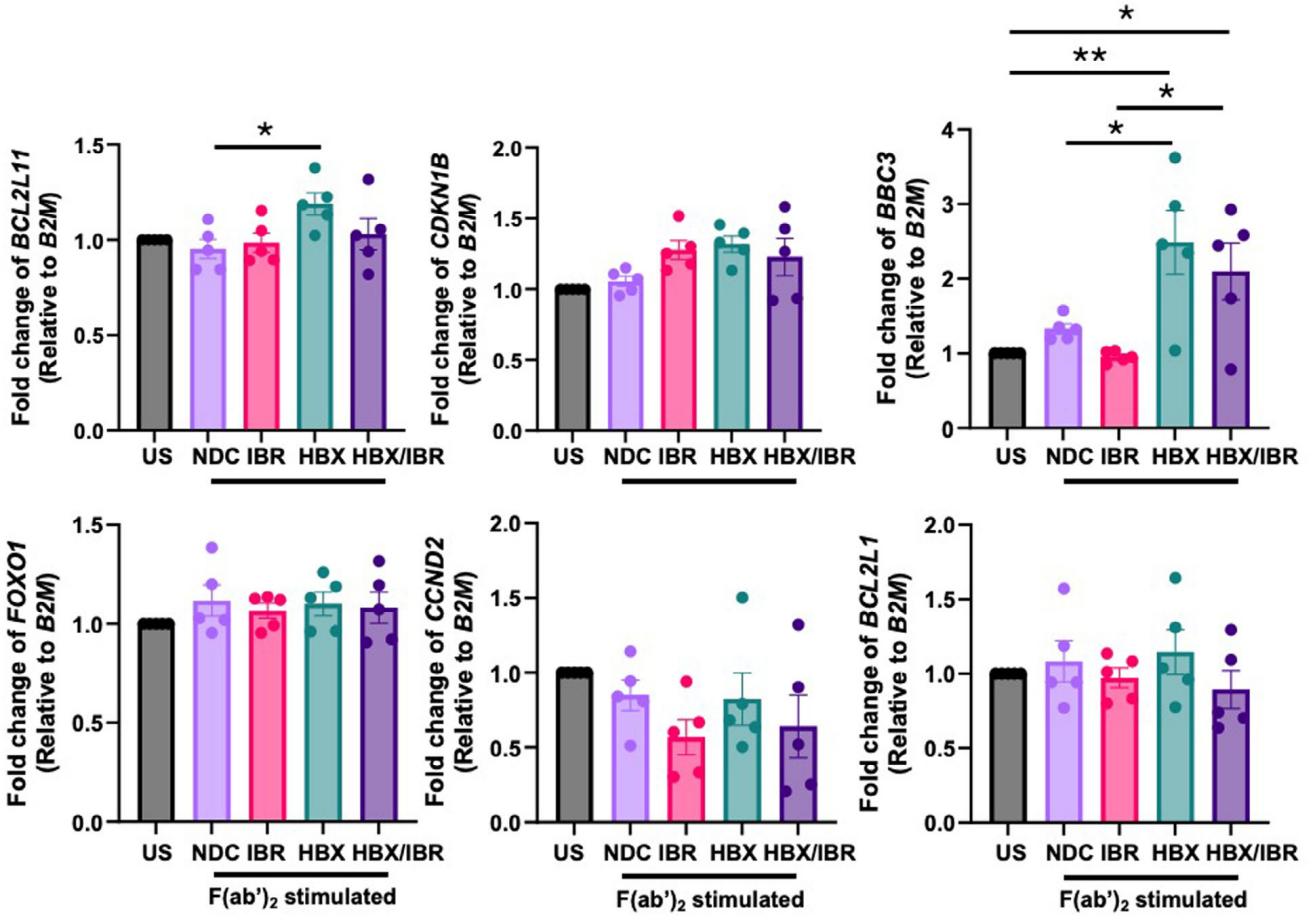
HBX19818 treatment of primary CLL cells increases specific pro‐apoptotic FOXO1 target genes. Primary CLL samples were BCR‐crosslinked (10 ng·mL^−1^) for 30 min or left unstimulated, after pre‐treatment with ibrutinib (IBR; 1 μm), HBX19818 (HBX; 8 μm) alone or in combination, or DMSO control for 1 h. Cells were stimulated for 24 h, then RNA/cDNA was generated. RT‐qPCR was used to assess transcription levels of *BCL2L11*, *CDKN1B*, *BBC3, FOXO1*, *CCND2, BCL2L1, CDKN1A* and *B2M* (reference gene) respectively in drug‐treated cells (*n* = 5) as indicated. Results are normalised to US/NDC control. Each dot represents an individual CLL sample. Data expressed as the mean ± SEM. Statistics calculated by unpaired *t*‐test; *P < 0.05, **P < 0.01.

Analysis of FOXO‐regulated apoptosis‐related genes *BBC3* (encoding PUMA) and *BCL2L11* (encoding BIM) revealed a significant upregulation of both genes with HBX19818 treatment in primary CLL cells, while combination with ibrutinib enhanced *BBC3* and *BCL2L11* expression further in MEC1 cells and maintained levels in primary CLL cells (Fig. 6; Fig. S14C,D). *CDKN1B* expression increased in response to HBX19818 treatment in primary CLL cells, supporting the significant upregulation of *CDKN1B* observed in MEC1 cells alone and in combination with ibrutinib (Fig. 6; Fig. S14E). Furthermore, there was a decrease in *CCND2* with ibrutinib alone in CLL models, which was significantly reduced in MEC1 cells. Interestingly, HBX19818 alone significantly increased the expression of both *CCND2* compared to untreated controls. The combination treatment resulted in a downregulation of *CCND2* to a similar level as with ibrutinib alone (Fig. 6; Fig. S14F). Finally, in MEC1 cells, both ibrutinib and HBX19818 treatments alone significantly increased *IGFR1* compared to untreated control MEC1 cells. The combination treatment further upregulated *IGFR1* compared to both single treatments (Fig. S14G). Overall, these findings support the role of USP7 inhibition in generating an anti‐proliferative response and upregulating FOXO1‐regulated transcription.

To determine whether the USP7‐mediated effects occurred in a FOXO1‐dependent manner, MEC1 cells were transduced with an shRNA lentiviral vector targeting *FOXO1*. FOXO1 gene and protein expression was significantly reduced using sh*FOXO1* construct 5 (sh*FOXO1*‐5) compared to SCR control and was used in all subsequent experiments (Fig. 7A; Table S4). As previously shown, ibrutinib did not alter the viability of either FOXO1 KD or SCR MEC1 cells. In contrast, HBX19818 alone and in combination with ibrutinib significantly reduced the viability of SCR cells, while drug sensitivity was reduced in FOXO1 KD cells (Fig. 7B). Proliferation assays also revealed a reduction in cell cycle arrest with HBX19818‐containing treatments in FOXO1 KD cells compared with SCR cells (Fig. 7C). Analysis of FOXO1 target genes revealed that *CCND2* levels were significantly lower in FOXO1 KD compared to SCR controls. In SCR cells, *CCND2* expression was reduced by IBR and the combination treatment, while it was significantly increased by HBX19818, similar to that noted previously (Fig. S14F). Although the pattern of *CCND2* expression was similar in FOXO1 KD cells, the changes did not reach significance (Fig. 7D). While *BCL2L11* expression was upregulated in SCR MEC1 cells following combination treatment, this did not reach significance as observed in USP7 KD MEC1 cells treated with IBR (Fig. 7D; Fig. S7E). Interestingly, FOXO1 KD elicited a reduction in *BCL2L11* with the combination treatment, albeit not significantly, suggesting a FOXO1‐dependent regulation (Fig. 7D). *CDKN1B* expression was significantly increased following combination treatment in SCR cells, while this significance was lost in FOXO1 KD cells (Fig. 7D). Collectively, these data indicate that pro‐apoptotic and anti‐proliferative effects of HBX19818 in MEC1‐CLL cells occur, at least in part, in a FOXO1‐dependent manner.

**Fig. 7:**
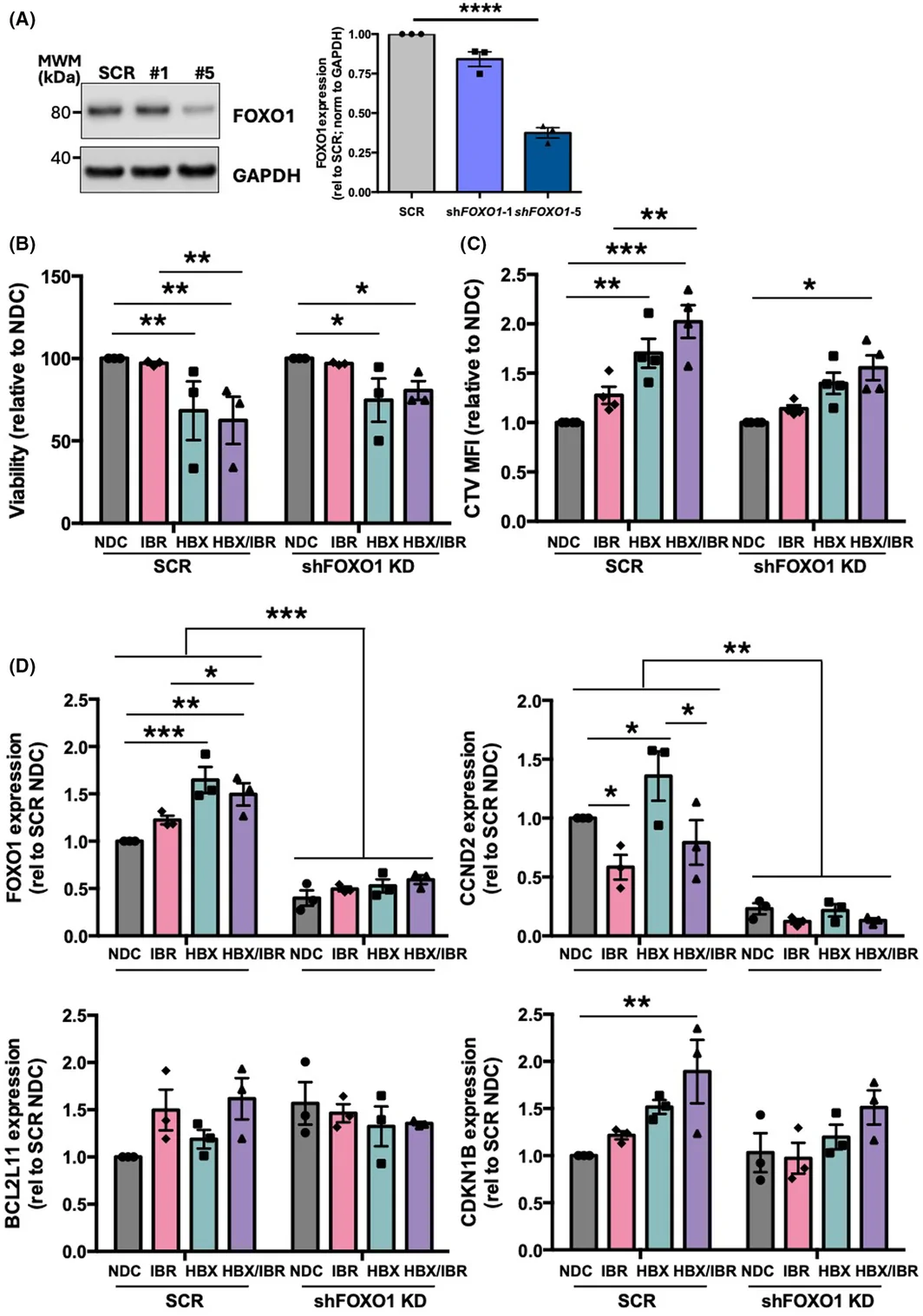
FOXO1 expression is required for HBX19818‐mediated reduction of MEC1 cell viability and proliferation. MEC1 cells were lentivirally transduced with a FOXO1‐targeting shRNA construct or SCR control. FOXO1 KD or SCR MEC1 cells were treated with IBR (1 μm), or HBX19818 (HBX; 10 μm), and combination (HBX/IBR) for 48 h (*n* ≥ 3). (A). Representative western blot of MEC1 transduced with shRNA‐*FOXO1*‐1 or shRNA‐*FOXO1*‐5 constructs or SCR control. The blots were probed with FOXO1 and GAPDH > 72 h post transduction. Relative protein levels of FOXO1 and SCR between *FOXO1* KD constructs and SCR MEC1 cells are shown (*n* = 3). shRNA‐*FOXO1*‐5 construct was used for the downstream analysis shown; (B). FOXO1 KD MEC1 cells were stained with Annexin V and 7‐AAD to assess cell viability following treatment with drugs as indicated for 48 h, or no drug control (NDC/DMSO). Viability is defined as Annexin V^neg^ and 7‐AAD/DAPI^neg^. Percentage viable cells for each condition are relative to NDC (*n* = 3); (C). FOXO1 KD MEC1 cells were stained with CTV to evaluate cell proliferation, following drug treatment as indicated for 48 h (*n* = 4), or NDC. Geometric means for each condition are relative to NDC. (D). FOXO1 KD MEC1 cells were treatment as indicated for 24 h, or NDC, then RNA/cDNA was generated. RT‐qPCR was used to assess transcription levels of *FOXO1*, *CCND2, BCL2L11, CDKN1B* and *B2M* (reference gene) respectively in MEC1 transduced with shRNA construct FOXO1 (*n* = 3) or SCR (*n* = 3) as indicated. Results are normalised to SCR‐NDC. Each dot represents a biological replicate. Data expressed as the mean ± SEM. Statistics calculated using one‐way ANOVA Tukey test, relative to unstimulated NDC; *P < 0.05, **P < 0.01, ***P < 0.001, ****P < 0.0001.

## 4 Discussion

In this study, we investigated the link between FOXO1 activity and the DUB protein USP7, and established that FOXO1 directly interacts with USP7 in primary CLL cells. This interaction enables the reduction of FOXO1 function, as inhibition of USP7 expression/activity partially reduced the transient FOXO1 upregulation. USP7 inhibition combined with ibrutinib enhanced FOXO1 nuclear localisation and increased FOXO1 activity, promoting transcription of FOXO target genes, while FOXO1 KD impaired the pro‐apoptotic and anti‐proliferative effects of HBX19818, and the modulation of FOXO target genes.

Transient elevation of FOXO1 expression during the early stages of BCR stimulation in CLL cells has not been reported in mature B cells [18]. Here, we demonstrated that targeting BTK with ibrutinib, as well as AKT or mTOR kinase with AZD5363 or AZD8055 respectively, decreased BCR‐induced upregulation of FOXO1, suggesting that FOXO1 is transiently stabilised in the cytoplasm of CLL cells following BCR‐mediated AKT activation [19]. FOXO family members can be regulated by ubiquitination, with studies demonstrating that USP7 inactivates FOXO via deubiquitylation and subsequent nuclear export in response to oxidative stress [21].

USP7, together with other DUB family members: USP8, USP9X, USP10 and USP14, is upregulated in primary CLL cells [22, 31]. Several studies have demonstrated that high levels of USP7 are directly associated with tumour progression in multiple myeloma (MM) [32], breast cancer [33], cervical cancer [34], ovarian cancer [35] and prostate cancer [36]. USP7 can modulate PI3K/AKT signalling through negative regulation of PTEN in CLL cells: targeted inhibition of USP7 with P5091 disrupted the USP7‐PTEN network promoting PTEN stabilisation and nuclear localisation enabling it to exert tumour suppressive functions [22]. USP7‐PTEN deregulation has also been reported in prostate cancer, with USP7 upregulation leading to elevated association with PTEN, predominantly residing in the cytoplasm [36]. The cytoplasmic localisation of PTEN mediated by USP7 interaction results in the removal of mono‐ubiquitination, a key regulator of PTEN nuclear access, thus enhancing PI3K‐AKT signalling.

The first target of USP7 was p53, responsible for promoting its stabilization [37]. It is now clear that USP7 also stabilises MDM2 under resting conditions [38], highlighting the dynamic regulation of the p53‐MDM2 network by USP7. In CLL cells, P5091 can induce apoptosis and inhibit the cell cycle of both *TP53*‐mutated MEC1 and *TP53* wild‐type EHEB cells, suggesting that the tumour suppressive effect of P5091 is independent of *TP53* mutational status in CLL [22, 26, 39]. Similarly, the USP7 inhibitor P22077 reduces the viability and proliferation of AML cells *in vitro* and *in vivo*, working independently of p53 mutational status [13]. However, the selective USP7 inhibitor FX1‐5303 showed more efficacy in inhibiting proliferation in *TP53* wild‐type compared to *TP53* mutated AML cell lines [14], indicating that p53 may play a cell‐specific role in mediating USP7‐dependent cell death.

In MEC1 cells, USP7 KD significantly enhanced FOXO1 nuclear translocation and activity in the presence of ibrutinib, indicating that USP7 contributes to the inhibition of FOXO1 transcriptional function. Due to the direct interaction between FOXO1 and USP7 in CLL cells, we propose that USP7 obstructs FOXO1 anti‐tumour activity by removing a mono‐ubiquitination mark on FOXO1 and preventing FOXO1 from effectively transcribing its target genes. While the direct role of USP7 in reducing FOXO1 activity through deubiquitination in CLL cells remains putative and a limitation of the current study, this mechanism is supported by studies showing that mono‐ubiquitination of FOXO4 upregulated its transcriptional activity, and USP7‐KD resulted in FOXO4 nuclear accumulation and activation [21]. This suggests that USP7 is involved in the suppression of FOXO activity. Gao *et al*. demonstrated through proteome analysis of USP7 KD melanoma cell lines that the PI3K/AKT pathway and FOXO are targets of USP7 deubiquitination activity [40]. While USP7 inhibitors alone demonstrated only a small effect on FOXO1 activity in CLL cells, the combined targeting of FOXO1 with BTK and USP7 inhibitors resulted in a greater effect on FOXO1 nuclear localisation, DNA‐binding activity and transcriptional activity. This combination may therefore maximise aspects of the anti‐tumour potential of FOXO1 in CLL cells, relating to the induction of anti‐proliferative and pro‐apoptotic genes. However, our data also demonstrate the upregulation of *IGFR1* in MEC1 cells targeted with ibrutinib and HBX19818 inhibition. Of note, *IGFR1* is a Foxo1 target that was induced in a mouse model of CLL resistance after long‐term treatment with the PI3Kδ inhibitor idelalisib, through upregulation of MAPK signalling [41]. Ondrisova *et al*. reported that long‐term ibrutinib treatment leads to FOXO1 upregulation leading to RICTOR/mTORC2 activation, leading to a distinct FOXO1‐mediated resistance mechanism [42]. These reports, coupled with the consideration that MEC1 cells lack p53 activity, indicate that the role of FOXO1 in CLL cells is dual‐faceted, with its function as a tumour‐suppressor or ‐promotor may be dependent on treatment duration, treatment experience of the cells and/or how aggressive the disease is, reflecting the complexity and heterogeneity of CLL.

Interestingly, while USP7 KD elevated p‐AKT^S473^ and p‐FOXO1^T24^ levels, these signals were reduced when MEC1 cells were treated with USP7 selective inhibitors. Furthermore, FOXO1 levels were also significantly elevated in the USP7 KD cells potentially due to the elevation in BCR‐associated signals. This may occur because reduced USP7 expression promotes redundancy between DUB family members. Supporting this, USP9x aids in mTORC2 assembly through deubiquitylation of Rictor to elevate AKT activation, demonstrating that DUB family members have the potential to interact with BCR‐mediated AKT signalling at multiple levels [43]. Indeed, we demonstrate the presence of USP9x in the FOXO1/USP7 interactome and may cooperate with USP7 to promote BCR signalling. To test the impact of their interaction more fully, performing USP7 and FOXO1 knockout and rescue experiments would have enabled a more specific investigation of their impact on CLL cells. However, we found that achieving a more efficient KD of USP7 had a negative impact on MEC1 cell survival (data not shown).

Considering the roles of DUBs in the UPS pathway and their involvement in a number of cancers, there has been growing interest in exploiting DUBs as therapeutic targets [44]. Numerous pre‐clinical studies have been performed, with pan‐DUB inhibitors and selective inhibitors of individual DUB proteins demonstrating efficacy in inducing apoptosis and inhibiting tumour growth, including those specifically targeting USP7 [22, 32, 44, 45, 46]. However, the translation of DUB inhibitors towards clinical trial is still in its early stages, and further research is required to improve their safety, specificity and efficacy. The first trial of a DUB inhibitor VLX1570, selective for USP14, in MM did show anti‐tumour effects; however, the trial was discontinued due to severe lung toxicity caused by increasing doses of VLX1570 [47].

The anti‐tumour effects of HBX19818 were tested *in vivo* using a MEC1 CLL murine xenograft model. Therapeutic doses of HBX19818 were well‐tolerated in mice, resulting in a reduction of tumour load [31]. Use of HBX19818 in combination with cyclophosphamide *in vivo* had a stronger effect than cyclophosphamide alone, highlighting the potential for USP7‐directed therapies in poor prognostic CLL patients. While clinical practice has largely moved away from chemotherapeutic treatments for CLL patients, our data show that HBX19818 combined with ibrutinib can enhance the anti‐tumour potency of ibrutinib. Multiple studies demonstrate the potential of USP7 inhibitors for clinical translation; however, no USP7 inhibitors have yet entered clinical trial, which may be due to the highly conserved structural features of the catalytic domain of DUB proteins causing cross‐inhibition [48].

## 5 Conclusion

Building on our previous findings that FOXO1 is negatively regulated by BCR signalling [16], this study demonstrates a direct interaction between FOXO1 and USP7 in primary CLL cells, leading to a suppression of FOXO1 function. Simultaneous targeting of BCR‐mediated and USP7 signalling promotes FOXO1‐dependent anti‐tumour activity demonstrated by increased nuclear localization, DNA binding activity and modulation of FOXO target genes. While the precise and complex regulatory mechanisms of FOXO1 by various DUBs require further investigation, our data provide compelling evidence that targeting DUB proteins in combination with ibrutinib represents a promising therapeutic strategy in CLL.

## Conflict of interest

The authors declare no conflict of interest.

## Author contributions

AMM, HA, MM, HGJ: conceptualization. HA, JH, MM, JC, AMM: Methodology. HA, JH, MM, LC, JC, AMM, AS, AMcC: investigation. AMM, HA: funding acquisition. AMM: project administration. AMM, HGJ: supervision. AMM, HA: writing – original draft. HA, JH, MM, LC, JC, AS, AMcC, HGJ, AMM: writing – review & editing.

## Supporting information


**Table S1.** CLL patient clinical characteristics.
**Table S2.** Preparation of Inhibitors for use in this study.
**Table S3.** Western blot and co‐immunoprecipitation (co‐IP) antibodies information.
**Table S4.** shRNA constructs.
**Table S5.** TaqMan Probes for RT‐qPCR.
**Fig. S1.** FOXO1 expression is upregulated in *ex vivo* primary CLL cells, with lower activity associated with poor prognostic subgroups. (A) Representative western blots to assess FOXO1 expression/activation in *ex vivo* PB‐derived CLL cells (*n* = 19) compared with healthy CD19^+^ cells from healthy donors (*n* = 6). Mirror blots were probed for p‐FOXO1^T24^ (80 kDa; blot A), FOXO1 (80 kDa, blot B) and GAPDH (37 kDa; loading controls A&B). Molecular weight marker (MWM) in kilodalton (kDa). The GAPDH blots shown are the same as Fig. 2B (blot A) and Fig. S5A (blot B). Densitometry from these blots and those in Fig. 1A are shown in Fig. S1B,C; (B) Relative protein expression of phospho‐FOXO^T24^ and total FOXO relative to GAPDH between B cell from healthy donors (Healthy CD19^+^; grey bars; *n* = 6) and CLL patient samples (CLL; *n* = 19) is shown; (C) Relative protein expression of phospho‐FOXO^T24^ and total FOXO relative to GAPDH (loading control) between primary CLL patient samples is shown, comparing good prognostic subsets (no reported 11q(del) or 17p(del); *n* ≥ 6) vs poor prognostic subsets (reported 11q(del) or 17p(del); *n* = 5). Each dot represents an individual sample. Data expressed as the mean ± SEM. Statistics calculated by unpaired *t*‐test; *P < 0.05, **P < 0.01, ***P < 0.001.
**Fig. S2.** FOXO1 protein expression is transiently upregulated upon BCR crosslinking. Primary CLL cells were stimulated with F(ab')_2_ fragments (10 ng·mL^−1^) for the indicated time (0.5–24 h) or left unstimulated (US). Protein and RNA was isolated for analysis. (A) Representative western blot of primary CLL sample (CLL173) is shown. Blots were probed for p‐AKT^S473^ (60 kDa), AKT (60 kDa), p‐FOXO1^T24^ (80 kDa), FOXO1 (80 kDa) and GAPDH (37 kDa; loading control; *n* = 4–9 individual patients). Relative protein expression of (B) p‐AKT^S473^ and AKT, and (C) p‐FOXO1^T24^ and FOXO1 is shown, relative to GAPDH (*n* = 4–9 individual patients as indicated by individual points); (D) qPCR of *FOXO1* expression in BCR‐crosslinked CLL cells across the time‐course shown, shown relative to reference gene *B2M*, and normalised to US (*n* = 3). Molecular weight marker (MWM) in kilodalton (kDa). Each dot represents an individual CLL sample. Data expressed as the mean ± SEM. Statistics calculated by paired *t*‐test; *P < 0.05, ***P < 0.001, ****P < 0.0001.
**Fig. S3.** The transient upregulation of FOXO1 protein expression upon BCR‐crosslinking is downregulated by inhibitors targeting BCR‐mediated signals including mTOR kinase and AKT in primary CLL cells. Primary CLL cells were treated with increasing concentrations of (A) AZD8055—an mTOR kinase inhibitor (10 nm, 100 nm, 1 μm, 10 μm) or (B) AZD5383—an AKT inhibitor (10 nm, 100 nm, 1 μm, 10 μm) for 1 h in the presence or absence of BCR‐crosslinking or left untreated. Protein was isolated for analysis. (A) Western blot representative of *n* = 3 individual patient samples treated with AZD8055 are shown. Blots were probed for p‐FOXO1^T24^, FOXO1 and GAPDH (loading control). (B) A Western blot of *n* = 1 patient sample treated with AZD5363 is shown. Blots were probed for p‐FOXO1^T24^, FOXO1 and GAPDH (loading control).
**Fig. S4.** FOXO1 protein expression is significantly downregulated by ibrutinib in MEC1 cells. MEC1 cells were treated with the BTK inhibitor, ibrutinib (IBR; 1 μm) for the indicated timepoints (1 h and 24 h) or left untreated no drug control (NDC; DMSO). Protein was isolated for analysis. (A) Representative western blot of treated MEC1 cells is shown. Blots were probed for p‐AKT^S473^ (60 kDa), AKT (60 kDa), p‐FOXO1^T24^ (80 kDa), FOXO1 (80 kDa) and GAPDH (37 kDa; loading control). Relative protein expression of (B) p‐AKT^S473^ and AKT, and (C) p‐FOXO1^T24^ and FOXO1 is shown, relative to GAPDH, and normalised to NDC for each timepoint (*n* ≥ 3); Each dot represents an individual biological replicate. Molecular weight marker (MWM) in kilodalton (kDa). Data expressed as the mean ± SEM. Statistics calculated by paired *t*‐test; *P < 0.05, ****P < 0.0001.
**Fig. S5.** Protein expression of selected DUBs in primary CLL cells. (A). Representative western blot to assess protein expression of USP9x in *ex vivo* PB CLL cells (*n* = 21) compared with healthy CD19^+^ cells from healthy donors (*n* = 7). The western blot was probed for USP9x (270 kDa), and GAPDH (37 kDa; loading control; same blot as shown in Fig. S1A–blot B); (B) Left–Protein expression of USP9x, relative to GAPDH, between healthy B cells (*n* = 7) and CLL patient samples (*n* = 21); Right–Protein expression of USP9x, relative to GAPDH, between healthy B cells and CLL patient samples separated into Binet stage; (C) Representative western blot to assess protein expression of selected DUBs in *ex vivo* PB CLL cells (*n* = 19) compared with healthy CD19^+^ cells from healthy donors (*n* = 7). The western blot was probed for USP8 (130 kDa), USP10 (110 kDa), USP14 (60 kDa), USP28 (135 kDa) and β‐ACTIN (45 kDa; loading control); D‐G. Left–Protein expression of USP8 (D), USP10 (E), USP14 (F), USP28 (G), relative to β‐ACTIN, between healthy B cells (*n* = 7) and CLL patient samples (*n* = 19); Right–Protein expression of USP8 (D), USP10 (E), USP14 (F), USP28 (G), relative to β‐ACTIN, between healthy B cells and CLL patient samples separated into Binet stage. Each dot represents an individual biological replicate. Molecular weight marker (MWM) in kilodalton (kDa). Data expressed as the mean ± SEM. Statistics calculated by unpaired *t*‐test; *P < 0.05 or one‐way ANOVA (Binet staging comparisons).
**Fig. S6.** USP7 knockdown leads to an upregulation in FOXO1 activity in MEC1 cells. MEC1 cells were lentivirally‐transduced with USP7‐78‐targeting shRNA constructs or scrambled (SCR) control. Densitometry of western blots of transduced MEC1 treated with ibrutinib (IBR; 1 μm) for 1 h or NDC, including those shown in Fig. 3C. Protein expression of p‐AKT^S473^/AKT (A; *n* = 9), p‐FOXO1^T24^/FOXO1 (B; *n* = 9), AKT (C; *n* = 9) and FOXO1 (D; *n* = 9) relative to β‐ACTIN. Each dot represents an individual MEC1 biological replicate. Data expressed as the mean ± SEM. Statistics calculated using one‐way ANOVA Tukey test; *P < 0.05, **P < 0.01, ***P < 0.001, ****P < 0.0001.
**Fig. S7.** USP7 KD in MEC1 cells modulates FOXO1 target genes. MEC1 cells were transduced with shRNA USP7‐78 KD or scrambled (SCR) control. Cells were treated with ibrutinib (IBR; 1 μm) or left untreated (NDC/DMSO) for 24 h, then RNA/cDNA was generated. (A–H) RT‐qPCR was used to assess transcription levels of *FOXO1*, *FOXO3*, *FOXO4*, *BCL2L1*, *BCL2L11*, *CDKN1B*, *IGF1R* and *CDKN1A* respectively in MEC1 transduced with shRNA construct USP7‐78 (*n* ≥ 3) or SCR (*n* ≥ 3) as indicated. Results are normalised to SCR‐NDC control. Each dot represents a biological replicate. Data expressed as the mean ± SEM. Statistics calculated by unpaired *t*‐test; *P < 0.05, **P < 0.01, ***P < 0.001.
**Fig. S8.** USP7 KD enhances ibrutinib effect in halting cell cycle progression. MEC1 cells transduced with shRNA USP7‐78 (oil green), or scrambled (SCR = black) were stained with PI for cell cycle analysis following treatment with ibrutinib (IBR; 1 μm), or NDC (MSO) for 72 h. Data are the same as shown in Fig. 3H. Quantification of DNA content of USP7‐78 KD (*n* = 10), or SCR (*n* = 10) in percentage (%) for cell cycle phase (A) G1/G0, (B) S and (C) G2/M. Data expressed as the mean ± SEM. Statistics calculated by one‐way ANOVA, Tukey test.
**Fig. S9.** DUB inhibitor treatment reduces cell viability in a concentration‐dependent manner. Cells (PBMCs, MEC1 and HG3 CLL cell lines, and primary CLL cells) were treated for 48 h with increasing concentrations of DUB inhibitors (A–C: PR‐619; D–F: P5091) or vehicle (DMSO) control. PBMCs from healthy donors were stained with anti‐CD19, anti‐CD4 and anti‐CD5 antibodies, to detect B and T cells respectively, then viability was measured by FACS using Annexin V FITC/7‐AAD staining (*n* ≥ 3). The IC_50_ was calculated for MNCs (black line), healthy B (pink line) and T (oil green line) cells (B, E). The IC_50_ was measured for CLL (blue line; B, E), and MEC1 (brown line; C, F) and HG3 (grey line; C, F). (G) Table summarising the calculated IC_50_ values for PR‐619 and P5091 in cell types indicated, measured by μm. Data expressed as the mean ± SEM. Statistics calculated by one‐way ANOVA, Tukey test (A, D); *P < 0.05, **P < 0.01, ***P < 0.001, ****P < 0.0001.
**Fig. S10.** HBX19818 treatment reduces cell viability in a concentration‐dependent manner. (A–C) Cells (PBMCs and MEC1) were treated for 48 h with increasing concentrations of HBX19818 or vehicle (DMSO) control. PBMCs from healthy donors were stained with anti‐CD19, anti‐CD4 and anti‐CD5 antibodies, to detect B and T cells respectively, then viability was measured by FACS using Annexin V FITC/7‐AAD staining (*n* ≥ 3). The IC_50_ was calculated for MNCs (black line), healthy B (pink line) and T (oil green line) cells (A, B). The IC_50_ was measured for MEC1 (brown line; C). (D) Table summarising the calculated IC_50_ values for HBX19818 in cell types indicated, measured by μm. Data expressed as the mean ± SEM.
**Fig. S11.** Increasing concentrations of PR‐619 increases global ubiquitin levels in MEC1 cells in a concentration‐dependent manner. Representative western blot of MEC1 cells, treated with increased concentration of PR‐619 (3 μm, 6 μm, 9 μm) for the indicated timepoints (1 h, 2 h, 4 h) or no drug control (NDC; DMSO). The blots were probed for Ubiquitin and β‐ACTIN (loading control). Relative levels of Ubiquitin between NDC (grey bar; *n* = 3) and increasing concentrations of PR‐619 (3 μm, 6 μm, 9 μm) for (1 h), (2 h) and (4 h) are shown. Samples were normalised to loading control β‐ACTIN. Each dot represented an Individual datapoint. Data expressed as the mean ± SEM. Statistics calculated by one‐way ANOVA, Dunnett test; *P ≤ 0.05, **P ≤ 0.01, ***P ≤ 0.001.
**Fig. S12.** USP7 inhibitors halt cell cycle progression. MEC1 cells stained with PI for cell cycle analysis by quantitation of DNA content following drug treatment. Data are the same as shown in Fig. 4C. (A–C) Cells were treated with ibrutinib (IBR; 1 μm), P5091 (P; 10 μm) and P/IBR, or NDC (DMSO) for 72 h (*n* = 4). Quantification of DNA content in percentage (%) for cell cycle phase (A) G0/G1, (B) S and (C) G2/M. (D–F) Cells were treated with IBR, HBX19818 (HBX; 10 μm), and HBX/IBR, or NDC for 72 h (*n* = 4). Quantification of DNA content (%) for cell cycle phase (D) G0/G1, (E) S, and (F) G2/M. Data expressed as the mean ± SEM. The IBR treatment data shown are the same in A–C and D–F respectively. Statistics calculated by one‐way ANOVA, Tukey test; *P ≤ 0.05, **P ≤ 0.01, ***P ≤ 0.001, ****P ≤ 0.0001.
**Fig. S13.** BCR crosslinking induced a significant and rapid upregulation of total FOXO1, which was reversed by ibrutinib and DUB inhibitors. (A) Representative western blot of a primary CLL sample (CLL196), unstimulated or stimulated with F(ab')_2_ fragments (10 ng·mL^−1^) for 0.5 h. Cells were pre‐treated with ibrutinib (IBR; 1 μm), PR‐619 (PR; 1.5 μm), or HBX19818 (HBX; 8 μm) or DMSO control for 1 h alone or in combination with IBR. The blots were probed for p‐AKT^S473^ (60 kDa), AKT (60 kDa), p‐FOXO1^T24^ (80 kDa), FOXO1 (80 kDa), USP7 (135 kDa) and β‐ACTIN (45 kDa; loading control). PR‐619 treatment: *n* = 5 except combination + BCR stimulation (*n* = 2). HBX treatment: *n* = 5 except HBX or combination + BCR stimulation (*n* = 3); (B) Densitometry of p‐AKT^S473^/AKT (left), p‐FOXO1^T24^/FOXO1 (middle) and FOXO1 (right) relative to β‐ACTIN is shown for BCR crosslinked cells in the presence and absence of IBR ± PR‐619; *n* = 2–5 as indicated (B), or IBR ± HBX19818; *n* = 3–5 as indicated. (C) Each dot represents an individual CLL patient sample. Molecular weight marker (MWM) in kilodalton (kDa). Data expressed as the mean ± SEM. Statistics calculated by paired *t*‐test; *P < 0.05, **P < 0.01, ***P < 0.001, ****P < 0.0001.
**Fig. S14.** FOXO1‐targeted gene expression was significantly modulated by the combination of ibrutinib and HBX19818 in MEC1 cells. MEC1 cells were treated with ibrutinib (IBR; 1 μm) or HBX19818 (HBX; 8 μm) alone or in combination or left untreated (NDC/DMSO) for 24 h, then RNA/cDNA was generated. (A–G) RT‐qPCR was used to assess transcription levels of *FOXO1*, *CDKN1B*, *BBC3*, *BCL2L11*, *CCND2* and *IGF1R* respectively in MEC1 cells (*n* = 3). Results are normalised to NDC control. Each dot represents a biological replicate. Data expressed as the mean ± SEM. Statistics calculated by unpaired *t*‐test; *P < 0.05, **P < 0.01, ***P < 0.001, ****P < 0.0001.

## Data Availability

Data sharing is not applicable to this article as no datasets were generated or analysed during this study.
