# Supplementary material for: USP7 inhibits FOXO1 activity in chronic lymphocytic leukaemia cells enhancing cell survival
Source: Mol Oncol. 2026 Oct 21:10.1002/1878-0261.70350. Online ahead of print. doi: 10.1002/1878-0261.70350 (PMC13685403; doi:10.1002/1878-0261.70350)
Supplement: Supplementary file 1 [file MOL2-9999-0-s001.docx]

**Supplemental Data File for:**

**USP7 inhibits FOXO1 activity in chronic lymphocytic leukaemia cells enhancing cell survival**

Hassan N.B. Almuhanna^1^, Jodie Hay^1^, Michael Moles^1^, Lucy Clarke^1^, Jennifer Cassels^1^, Alison Sefcick^2^, Alison M. McCaig^1,3^, Heather G. Jørgensen^1^, Alison M. Michie^1^

^1^School of Cancer Sciences, College of Medicine, Veterinary and Life Sciences,

University of Glasgow, Glasgow UK; ^2^NHS Greater Glasgow & Clyde Royal Alexandra Hospital, Paisley, UK; ^3^NHS Greater Glasgow & Clyde Queen Elizabeth University Hospital, Glasgow, UK.

| CLL8 | F | A | UT | del(11q) |
| --- | --- | --- | --- | --- |
| CLL9 | F | A | T | - |
| CLL57 | M | C | UT | - |
| CLL78 | M | - | T | del(17p) |
| CLL93 | M | C | T | del(17p) |
| CLL102 | F | C | T | del(11q) |
| CLL109 | M | B | - | del(17p) |
| CLL116 | M | A | UT | Normal |
| CLL122 | M | - | T | del(17p) |
| CLL132 | F | B | UT | del(17p) |
| CLL138 | F | A | T | - |
| CLL140 | F | C | UT | T12 |
| CLL147 | M | C | UT | Normal |
| CLL151 | M | B | UT | del(11q) |
| CLL162 | M | - | - | - |
| CLL163 | M | - | - | - |
| CLL168 | M | B | UT | T12 / del(13q) |
| CLL169 | F | C | UT | del(11q) |
| CLL170 | F | - | T | - |
| CLL171 | M | C | UT | - |
| CLL172 | M | - | - | Normal |
| CLL173 | M | B | T | del(11q) |
| CLL175 | M | C | T | - |
| CLL176 | M | A | UT | - |
| CLL177 | M | B | UT | - |
| CLL179 | M | B | T | - |
| CLL180 | F | B | T | - |
| CLL184 | M | - | T | - |
| CLL185 | M | C | - | - |
| CLL186 | M | C | UT | - |
| CLL187 | M | B | UT | - |
| CLL189 | M | B | T | - |
| CLL190 | M | C | UT | - |
| CLL191 | M | A | UT | del(11q) / del(17p) |
| CLL193 | F | A | UT | - |
| CLL194 | M | C | UT | - |
| CLL195 | M | C | UT | - |
| CLL196 | F | B | UT | - |
| CLL198 | M | - | T | - |
| CLL200 | M | C | - | - |
| CLL224 | F | B | UT | del(11q) |

Table S1. CLL patient clinical characteristics.

^a^ Binet staging: Stage A = mild, B = intermediate, C = advanced; ^b^ If previously undergone treatment, it was not within three months of sample collection; T = Treated, UT = Untreated; - = not determined. Normal = no cytogenetic abnormality detected by FISH. *IGHV* mutational status was not determined in these CLL samples.

| Inhibitor | Stock Concentration | M.Wt^a^ | Diluent | Working conc. for primary CLL cells^b^ | Working conc. for CLL cell lines^b^ |
| --- | --- | --- | --- | --- | --- |
| Ibrutinib | 10 mM | 440.5 | DMSO | 1 µM | 1 µM |
| P5091 | 10 mM | 348.22 | DMSO | 2 µM | 10 µM |
| HBX19818 | 10 mM | 421.96 | DMSO | 8 µM | 10 µM |
| PR-619 | 50 mM | 223.28 | DMSO | 2 µM | 3 µM |

**Table S2: Preparation of Inhibitors for use in this study.**

^a^ Molecular Weight; ^b^ Concentration of inhibitors used in the experiments, unless otherwise stated.

| Specificity | Species | Dilution | Diluent | Cat. No. | Manufacturer |
| --- | --- | --- | --- | --- | --- |
| AKT | **Rabbit** | **1:2000** | **5% BSA** | **9272** | **CST** |
| p-AKT (Ser473) | **Rabbit** | **1:2000** | **5% BSA** | **4060** | **CST** |
| β-Actin* | **Rabbit** | **1:1000** | **5% BSA** | **3700** | **CST** |
| FOXO1^#^ | **Rabbit** | **1:1000** | **5% BSA** | **2880** | **CST** |
| p-FOXO1 (Thr24) | **Rabbit** | **1:1000** | **5% BSA** | **9464** | **CST** |
| GAPDH* | **Rabbit** | **1:1000** | **5% Milk** | **5174** | **CST** |
| HAUSP/USP7^#^ | **Rabbit** | **1:1000** | **5% BSA** | **4833** | **CST** |
| Lamin A/C* | **Rabbit** | **1:1000** | **5% BSA** | **2032** | **CST** |
| β-Tubulin* | **Rabbit** | **1:1000** | **5% BSA** | **2128** | **CST** |
| USP8 | **Rabbit** | **1:1000** | **5% BSA** | **8728** | **CST** |
| USP9x | **Rabbit** | **1:1000** | **5% Milk** | **14898** | **CST** |
| USP10 | **Rabbit** | **1:1000** | **5% Milk** | **5553** | **CST** |
| USP14 | **Rabbit** | **1:1000** | **5% BSA** | **11931** | **CST** |
| USP28 | **Rabbit** | **1:1000** | **5% BSA** | **4217** | **CST** |
| Rabbit IgG control^†^ | Rabbit | - | IP lysis buffer | ab37415 | Abcam |
| Anti-rabbit goat IgG, HRP-linked | Rabbit | 1:1000 | 5% Milk | 7074 | CST |
| Anti-mouse horse IgG, HRP-linked | Mouse | 1:1000 | 5% Milk | 7076 | CST |
| IRDye®680RD Goat-anti Rabbit IgG | Rabbit | 1:15000 | TBST | 926-68071 | Li-Cor |
| IRDye®800CW Goat-anti Mouse IgG | Rabbit | 1:15000 | TBST | 827-08364 | Li-Cor |

**Table S3: Western blot and co-immunoprecipitation (co-IP) antibodies information.**

*Protein loading controls. ^#^These antibodies were used for immunoblotting and co-IP. ^†^This antibody was used in co-IP as a rabbit IgG control.

| **Target** | **Clone ID** | **Target sequence** | **Vector ID** | **Levels in KD MEC1** | **Region** |
| --- | --- | --- | --- | --- | --- |
| USP7 | TRCN0000004057 | CCAGCTAAGTATCAAAGGAAA | TRC1 | RNA: 25%  Protein: 88% | CDS |
| USP7 | TRCN0000010845 | CGTGGTGTCAAGGTGTACTAA | TRC1 | RNA: 53%  Protein: 62% | CDS |
| USP7 | TRCN0000318521 | CCTGGATTTGTGGTTACGTTA | TRC2 | RNA: 63%  Protein: 55% | CDS |
| USP7 | TRCN0000318578 | CCAGCTAAGTATCAAAGGAAA | TRC2 | RNA: 41%  Protein: 58% | CDS |
| FOXO1 | TRCN0000039578 | GCCTGTTATCAATCTGCTAAA | TRC1 | RNA: 41%  Protein: 34% | 3’ UTR |

**Table S4: shRNA constructs.**

All shRNA constructs were purchased from Sigma. Knock-down (KD) levels indicate the amount of USP7 RNA/protein expression remaining.

| Gene | Species | Chromosome Location | Assay ID |
| --- | --- | --- | --- |
| B2M | Human | Chr.15: 44711487 - 44718159 | Hs00984230_m1 |
| BBC3 | Human | Chr.19: 47220822 - 47232998 | Hs00248075_m1 |
| BCL2L1 | Human | Chr.20: 31664452 - 31723999 | Hs00236329_m1 |
| BCL2L11 | Human | Chr.2: 111120914 - 111168445 | Hs00153408_m1 |
| CCND2 | Human | Chr.12: 4273736 - 4305356 | Hs00153380_m1 |
| FOXO1 | Human | Chr.13: 40555664 - 40666597 | Hs01054576_m1 |
| FOXO3 | Human | Chr.6: 108559823 - 108684769 | Hs00818121_m1 |
| FOXO4 | Human | Chr.X: 71096149 - 71103534 | Hs00936217_g1 |
| GADD45A | Human | Chr.1: 67685177 - 67688338 | Hs00169255_m1 |
| GAPDH | Human | Chr.12: 6534405 - 6538375 | Hs99999905_m1 |
| IGF1R | Human | Chr.15: 98648539 - 98964530 | Hs00609566_m1 |
| SESN3 | Human | Chr.11: 95165513 - 95232541 | Hs00914870_m1 |
| USP7 | Human | Chr.16: 8892094 - 8963912 | Hs00931763_m1 |

**Table S5: TaqMan Probes for RT-qPCR.**

All primers/probes were purchased from ThermoFisher Scientific.
